# Extracellular Vesicles in Malaria: Shedding Light on Pathogenic Depths

**DOI:** 10.1021/acsinfecdis.3c00649

**Published:** 2024-02-06

**Authors:** Sangita Dey, Salini Mohapatra, Manoj Khokhar, Sana Hassan, Rajan Kumar Pandey

**Affiliations:** † CSO Department, Cellworks Research India Pvt Ltd, Bengaluru 560066, Karnataka, India; ‡ Department of Biotechnology, Chandigarh University, Punjab 140413, India; § Department of Biochemistry, All India Institute of Medical Sciences Jodhpur, Rajasthan 342005, India; ∥ Department of Life Sciences, Manipal Academy of Higher Education, Dubai 345050, United Arab Emirates; ⊥ Department of Medical Biochemistry and Biophysics, Karolinska Institute, Stockholm 17177, Sweden

**Keywords:** Extracellular vesicles, Malaria, Parasite communication, Biomarkers, Therapeutic targets

## Abstract

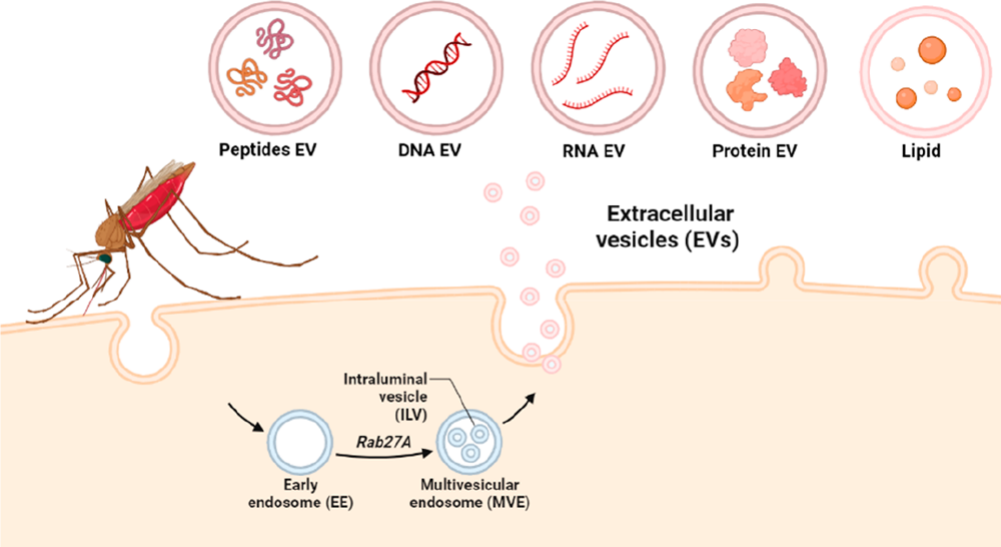

Malaria, a life-threatening infectious disease caused by *Plasmodium falciparum*, remains a significant global health challenge, particularly in tropical and subtropical regions. The epidemiological data for 2021 revealed a staggering toll, with 247 million reported cases and 619,000 fatalities attributed to the disease. This formidable global health challenge continues to perplex researchers seeking a comprehensive understanding of its pathogenesis. Recent investigations have unveiled the pivotal role of extracellular vesicles (EVs) in this intricate landscape. These tiny, membrane-bound vesicles, secreted by diverse cells, emerge as pivotal communicators in malaria’s pathogenic orchestra. This Review delves into the multifaceted roles of EVs in malaria pathogenesis, elucidating their impact on disease progression and immune modulation. Insights into EV involvement offer potential therapeutic and diagnostic strategies. Integrating this information identifies targets to mitigate malaria’s global impact. Moreover, this Review explores the potential of EVs as diagnostic biomarkers and therapeutic targets in malaria. By deciphering the intricate dialogue facilitated by these vesicles, new avenues for intervention and novel strategies for disease management may emerge.

Malaria, a deadly infectious disease, significantly burdens the global healthcare system, mainly in tropical regions along with subtropical parts of the world. As per the World Malaria Report 2022, malaria cases reached 247 million in 2021, slightly higher than the 245 million cases reported in 2020.^1^ Concerning malaria-related fatalities, there were approximately 619,000 deaths in 2021, which is somewhat lower than the 625,000 deaths reported in 2020. During the 2020–2021 pandemic, COVID-19 contributed to approximately 13 million additional malaria cases and 63,000 additional malaria-related deaths.^1^ In the year 2021, the WHO African region bore a substantial portion of the worldwide malaria burden, comprising roughly 95% of all reported malaria cases and 96% of malaria-related fatalities. Almost 80% of malaria-related deaths were in children under five years of age in this region. Among all nations, Nigeria (31.3%), the Democratic Republic of the Congo (12.6%), the United Republic of Tanzania (4.1%), as well as Niger (3.9%) were the homes to slightly over half of the global malaria deaths.^1^*Plasmodium*, a protozoan parasite, is the causal organism for this disease; however, *Plasmodium falciparum* occupies the highest virulence ranking among other *Plasmodium* species. Malaria transmission occurs in humans by the bite of pre-infected female *Anopheles* mosquitoes.^2^ Malaria symptoms include fever, chill, headache, fatigue, muscle pain, organ failure, and fatality in severe instances. Severe malaria has additional complications like cerebral malaria, severe anemia, severe lung dysfunction, and simultaneous malfunction of multiple vital organs.

## Life Cycle of Malaria Parasite

The life cycle of a malaria parasite is intricate, entailing interactions with an *Anopheles* mosquito and a vertebrate host. Initially, a female mosquito from the genus *Anopheles* bites a healthy individual and transfers sporozoites to the human skin through saliva. Once these sporozoites enter the bloodstream and invade hepatocytes, initiating asexual replication.^3^ During this hepatic stage, the infected hepatocytes rupture, leading to the release of numerous merozoites. Certain merozoites develop into dormant hypnozoites in specific instances of *P. vivax* and *P. ovale* infections. These hypnozoites persist within hepatocytes for extended periods, ranging from several months to up to four years, before becoming active and multiplying for a new phase of erythrocytic infection.^4^ This new infection phase involves merozoites interacting with RBCs. Merozoites attach and deform the host cell membrane surface, subsequently entering the RBCs for the second asexual reproduction through parasite-induced reorganization of the erythrocyte cytoskeleton.^5^ The *P. vivax* and *P. ovale* target younger erythrocytes, while *P. falciparum* and *P. knowlesi* invade erythrocytes of any age. In contrast, *P. malariae* exhibits a preference for aging or senescent erythrocytes. Following their invasion of RBCs, merozoites undergo replication to form trophozoites and subsequently schizonts. These schizonts rupture the RBCs, liberating merozoites, which then invade fresh RBCs, thus perpetuating the cycle of asexual replication.^5^

The malaria sexual reproduction cycle begins when certain trophozoites mature into male and female sexual progeny called gametocytes.^6^ These gametocytes play a crucial role in transmitting malaria infection from the mammalian host to the mosquito. Once an *Anopheles* mosquito bites a diseased host, mature gametocytes are ingested and transferred into the mosquito’s midgut. Here, the gametocytes transform into fertile gametes, leading to the subsequent stage where zygotes are converted into mobile and invasive ookinetes.^7^ These ookinetes, in turn, develop into oocysts in the midgut basal lamina. When the oocysts reach maturity, they flush out sporozoites, which move to the mosquito’s salivary gland. Subsequent mosquito bites to another healthy mammalian host lead to the transmission of the parasite, perpetuating the cycle.^8^

## Immune Pathogenesis of Acute and Severe Malaria

Utilizing genome-wide expression analysis, recent research has investigated the immune responses during spontaneous *P. falciparum* infection in malaria-endemic environments. These investigations showed that acute malaria is associated with an increase in gene expression profiles linked to neutrophil and erythroid-related cell activities.^9^ When comparing acute malaria to convalescent controls, higher expression of genes encoding Toll-like receptors (TLRs), TLR-signaling proteins, and components of interferon signaling pathways was seen. Furthermore, genes related to MAP kinase signal transduction pathways, apoptosis, and immune modulation were elevated in response to a natural *P. falciparum* infection.^9^ By utilizing comprehensive systems immunology tools in well-powered, longitudinal cohort studies, future research should build on current efforts.

Infections caused by *P. falciparum* and *P. vivax*, which are specific to humans, as well as zoonotic infections caused by *P. knowlesi*, are commonly observed and are linked to severe malaria, characterized by anemia, multiorgan failure, metabolic acidosis as well as cerebral malaria.^10^ This phenomenon was observed in the initial studies of malaria in both humans and mice, where researchers primarily investigated EVs originating from various sources, including platelets (PEVs), monocytes (MEVs), red blood cells (REVs), endothelial cells (EEVs), and lymphocytes (LEVs).^11^ In models of cerebral malaria, researchers commonly utilize two distinct species of the *Plasmodium* parasite: *P. yoelii*(12) and *P. berghei*,^13^ with a particular focus on *P. berghei* ANKA (PbA). In the early stage of infection, mouse strains that are prone to developing cerebral malaria (such as CBA/J, C57BL/6, and DBA1) exhibit elevated levels of plasma microvesicles, which is consistent with observations made in humans.^12−15^ Couper and colleagues made a noteworthy discovery regarding *P. berghei*-infected red blood cell-derived extracellular vesicles (pb-iREVs). The pb-iREVs harbored unique parasite content and, when studied *in vitro*, prompted the release of tumor necrosis factor (TNF) from macrophages, particularly via a TLR-4/MyD88-dependent pathway.^11,16^ Interestingly, pb-iREVs proved to be significantly more potent in activating macrophages when compared to viable *P. berghei*-infected red blood cells (iRBCs). Notably, the highest immunogenic pb-iREV levels coincided with the emergence of clinical symptoms and were accompanied by the most immunogenic pb-iREVs. The researchers concluded that the increased presence of pb-iREVs, characterized by a unique pro-inflammatory profile, develops severe malaria.^16^ Mantel and colleagues demonstrated that, whereas pf-iREVs can activate neutrophils, human monocytes are the primary target immune cells.^11,17^

## Extracellular Vesicles and Their Function in Intercellular Communication

**Figure 1:**
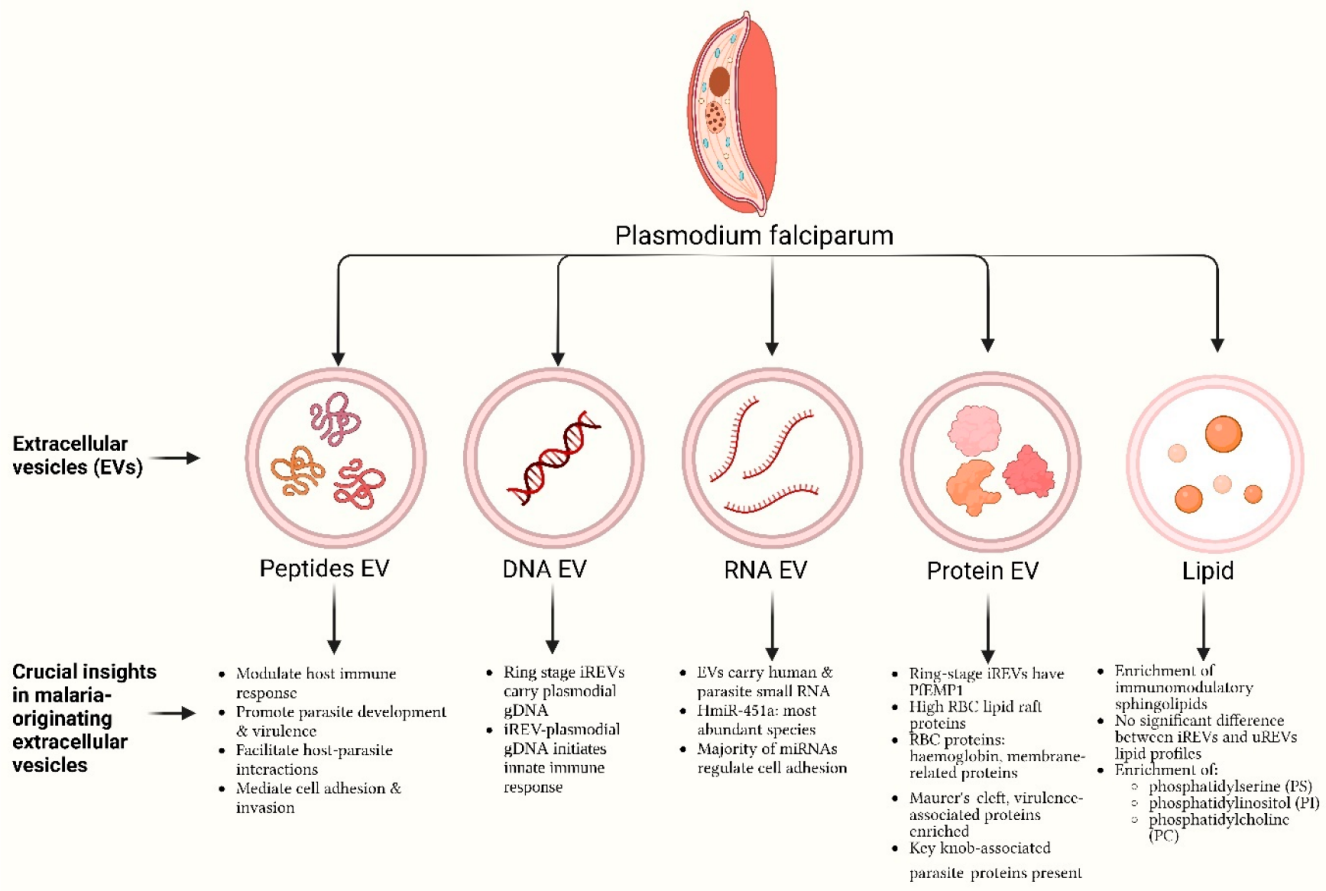
Extracellular vesicles (EVs) with diverse cargoes—Peptide EVs, DNA EVs, RNA EVs, Protein EVs, and Lipid EVs—play pivotal roles in *Plasmodium*-infected malaria. These EVs mediate complex interactions, influencing immune responses and disease progression.

**Figure 2:**
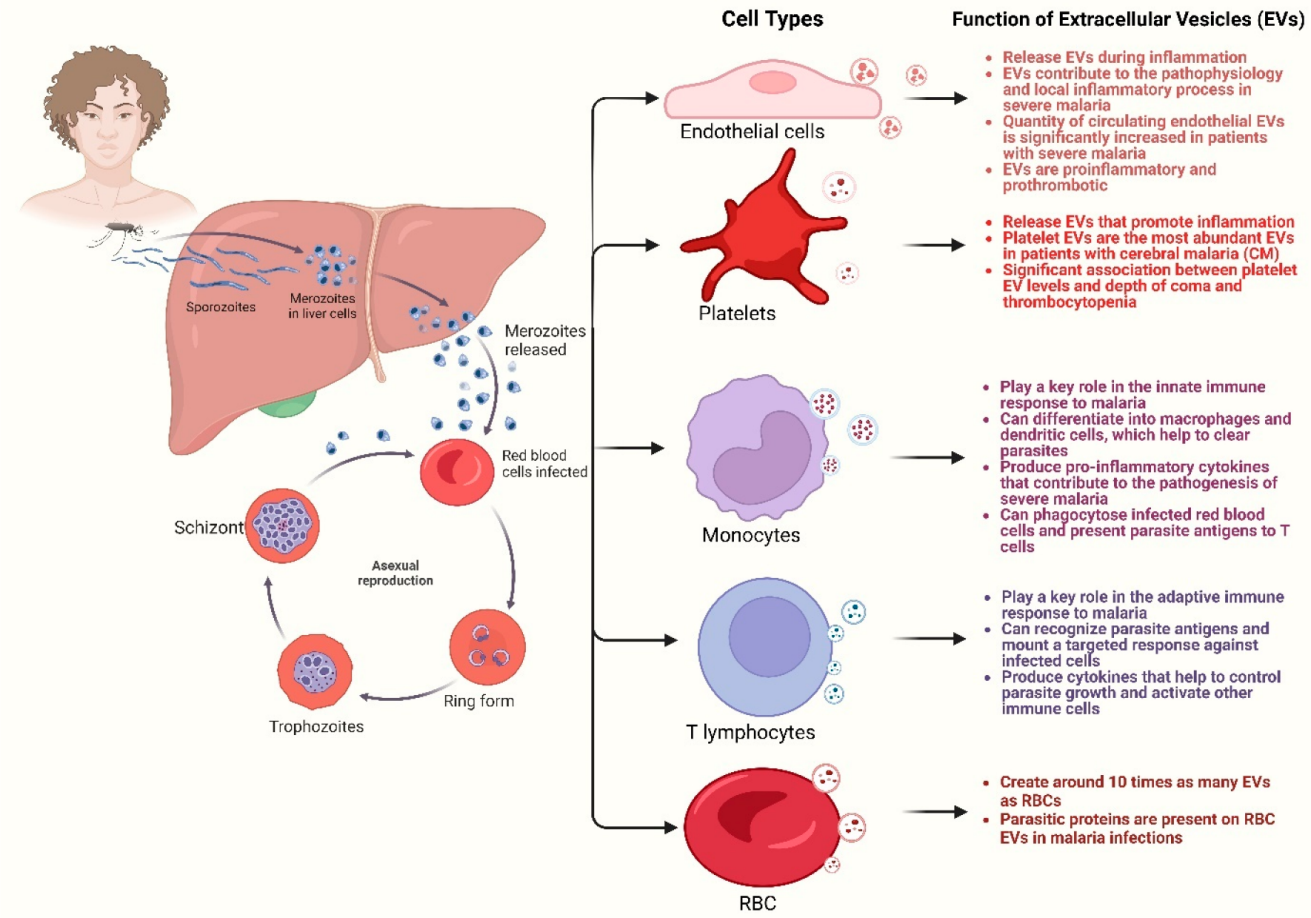
Extracellular vesicles (EVs) are key mediators in malaria, facilitating intricate interactions between *Plasmodium* parasites and the host immune system. EVs, such as exosomes and microvesicles, released by infected cells impact various immune cells. This figure legend highlights their roles in endothelial cell activation, platelet-related thrombosis, monocyte-driven immune responses, T lymphocyte regulation, and red blood cell-mediated parasite dissemination. Understanding EV molecular cargo and balancing pro/anti-inflammatory signals is crucial for deciphering their precise contributions, offering potential therapeutic and diagnostic avenues in ongoing malaria research.

Cells employ diverse mechanisms of communication to ensure proper tissue development and functioning. Traditional modes of cell communication include tight junctions, adhesion molecules, and soluble factors that act locally or in an endocrine manner.^18^ In addition to these well-established methods, a recently acknowledged mode of cell communication is through EVs, which were formerly thought of as cellular waste disposal units. This discovery has greatly expanded our knowledge of cellular communication. EVs significantly facilitate cell-to-cell communication, which enables the exchange of materials and information between cells (Figure 1). EVs possess the ability to directly activate recipient cells by working as signaling complexes. For example, macrophages and neutrophils-derived EVs bind to platelets and trigger platelet activation (Figure 2). Additionally, EVs can facilitate the transfer of receptors between cells.^19^

## Biogenesis and Release of EVs: Exosomes, Microvesicles, and Apoptotic Bodies

EVs encompass a heterogeneous group of membrane-bound vesicles that have their origins in either the endosome or the cell membrane. It was the pioneering work by Pan and Johnstone (1983) that provided one of the earliest descriptions of EVs.^20^ Their early research revealed that EVs act as a crucial component of a cellular disposal system that removes undesirable components. Over time, subsequent investigations have unveiled the significance of EVs in intercellular communication, influencing various physiological processes and the progression of pathological conditions.^21^

EVs can be categorized as per their release mechanism or size. If they are dispersed by the plasma membrane’s outward budding, known as shedding microvesicles or ectosomes.^22^ Another release mechanism involves inward endosomal membrane budding, forming multivesicular bodies (MVBs), and subsequent fusion of outer MVBs membrane with the plasma membrane releases exosomes.^20^ In addition to variation in release mechanisms, vesicle size is employed as a means of classification. Shedding MVBs’ diameter typically ranges from 50 to 10,000 nm, while exosomes have the same from 30 to 150 nm.^20^ Overall, EVs encompass a diverse range of vesicles. The size of these vesicles ranges from 30 to 1000 nm. The detailed biogenesis of EVs is mentioned below.

### Exosomes

Since it was discovered, significant advancements have been made in comprehending the biogenesis of EVs. Exosomes are made up of three different compartments and come from the endosomal network. These are early endosomes, recycling endosomes, and late endosomes. Early endosomes incorporate contents from endocytic vesicles for recycling, degradation, or exocytosis. Recycling endosomes leads to the sorting of early marked materials for recycling. The remaining early endosomes transform into late endosomes, where 30–100 nm vesicles bud within their lumen to house contents for degradation or export selectively. Late endosomes, also known as MVBs, due to the presence of multiple small vesicles,^23^ can fuse with lysosomes for content breakdown or merge with the plasma membrane, secreting 30–100 nm vesicles called exosomes into the extracellular space.

To gain insight into exosome formation, we first need to explore the primary mechanism behind intraluminal vesicle (ILV) formation. Various literature suggests that ILV formation consists of two distinct processes. Initially, the endosome membrane is reorganized into specialized units that are heavily loaded with certain membrane proteins called tetraspanins, which possess four transmembrane domains.^24^ These specialized units are known as tetraspanin-enriched microdomains (TEMs), forming a unique tertiary structure.^25^ It is believed that TEMs bring together the proteins necessary for ILVs formation through these interactions. Two tetraspanins namely CD9 and CD63 serve as exosome markers and are targeted for exosome isolation due to their role in exosome formation.^26^ CD63 is also involved in various other processes, including apoptosis in neutrophils,^27^ platelets,^28^ Weibel Palade bodies in vascular endothelium,^29^ and lysosome-related vesicles in leukocytes, such as T lymphocytes,^30^ eosinophils,^31^ mast cells,^32^ basophils,^33^ and megakaryocytes.^29^

The **e**ndosomal **s**orting **c**omplex **r**equired for **t**ransport (ESCRT) is a group of complexes that are involved in the second step of exosome production. Four distinct multiprotein complexes are involved in ILVs formation, namely ESCRT-0, I, II, and III.^34^ The early endosome membranes are characterized by a high concentration of phosphatidylinositol 3-phosphate (PIP3). The ubiquitinated cargos, curved membrane topology, and this PIP3 all contribute to the recruitment of ESCRT-I as well as ESCRT-II. This drives the budding of the membrane while ESCRT-III completes the budding process. Alix, a protein, facilitates the recruitment of ESCRT-III to the ESCRT-1 and II sites by binding to both the TSG101 component of ESCRT-I and CHMP4 of ESCRT-III.^35^ TSG101 and Alix are also used as markers for exosomes.^36^

### Microvesicles

The process of microvesicle biogenesis also referred to as ectosomes, differs from exosome formation, which occurs through direct outward budding and plasma membrane fission. Microvesicles are generally larger and their size ranges from 50 to 2000 nm. Microvesicle formation relies on the interplay between phospholipid redistribution and cytoskeletal protein contraction. The translocation of phosphatidylserine to the outer membrane leaflet induces the membrane budding and vesicle formation.^37^ Actin–myosin interactions cause the cytoskeletal structures to contract, concluding this process.^38^ As per the literature, when checked in the melanoma model, overexpression of the GTP-binding protein ADP-ribosylation factor 6 (ARF6) leads to the increased production of microvesicles.^39^

### Apoptotic Bodies

Apoptosis is a controlled cellular mechanism for programmed cell death, which encompasses several stages including nuclear chromatin condensation, membrane blebbing, and the generation of apoptotic bodies or apoptosomes. These apoptosomes are specialized membrane-enclosed vesicles that contain cellular contents and play a distinct role in apoptosis.^40^ Apoptotic bodies typically have a larger size, ranging from 500 to 4000 nm, and contain organelles within the vesicles.^41^ Additionally, smaller vesicles in the 50–500 nm range are released during apoptosis; however, it is unclear if the apoptotic process caused these smaller vesicles to bleb during the apoptotic process.^42^ Existing data indicate that actin–myosin interactions play a role in mediating membrane blebbing.^43^

During normal development, macrophages phagocytose and locally clear most apoptotic bodies.^41^ This clearance procedure depends on particular interactions between phagocyte recognition receptors and modifications to the apoptotic cell membrane.^44^ One well-documented alteration entails the relocation of phosphatidylserine to the outer lipid layer, subsequently binding with Annexin V, a recognition signal for phagocytes. Another recognized modification in the membrane involves surface molecule oxidation, forming attachment sites for the complement protein C3b or thrombospondin. Following binding, these thrombospondins and C3b molecules are identified by receptors on phagocytes, effectively serving as markers for apoptotic bodies. Thrombospondin, Annexin V, as well as C3b, are widely accepted markers for apoptotic bodies.^45^

## Contents of EVs: Proteins, Nucleic Acids, Lipids, and Other Molecules

The contents of EVs vary based on their biogenesis, cell type, and physiological conditions. Usually, all EVs contain a diverse array of lipids, proteins, as well as nucleic acids. The loading of various types of cargo is conditional and depends on the vesicle type and the specific cell type it originated from. Substantial research efforts have been dedicated to characterizing the contents of EVs, resulting in the establishment of publicly available databases such as Vesiclepedia (http://www.microvesicles.org/),^46^ Exocarta (http://www.exocarta.org/),^47^ and EVpedia (https://evpedia.info/evpedia2_xe/).^48^ These databases include information on the protein, nucleic acid, and lipid content of EVs, along with information about the isolation and purification techniques employed. The details are mentioned below.

### Protein Contents

Various studies have been comprehensively analyzing the protein cargo of EVs, characterizing the vesicle contents.^49^ However, due to variations in separation techniques, diverse cell types, and culture conditions used for protein analysis, providing a conclusive perspective on the protein composition of different vesicle types is challenging. The common proteins found in EVs are proteins associated with biogenesis mechanisms, such as those linked to endosomal pathways, including components of the ESCRTs like ALIX and TSG101. Other frequently detected proteins, such as RAB11B, RAB27A, and ARF6, are also implicated in EV generation and release.^50^ Furthermore, EVs encompass a range of tetraspanins including CD9, CD63, and CD81, in addition to proteins associated with signal transduction (such as EGFR), antigen presentation, and various transmembrane proteins like LAMP1 and TfR. In general, EVs do not contain proteins associated with ER, Golgi, and nucleus. However, transcription factors like Notch and Wnt, which are typically located in the nucleus, have been detected inside EVs.^51^ Differences in data sets and analytical techniques employed for vesicle content studies underscore the need for standardization in both isolation and analysis methods. This standardization is crucial to obtain a clearer understanding of the protein composition in various EV subtypes and the signaling mechanisms that result in protein enrichment within EVs.

### Lipid Content

Apart from proteins, there has been comprehensive research conducted on the lipid composition of EVs in various contexts. Numerous studies have demonstrated that specific lipids can exhibit unique associations with different types of EVs. Illustrative examples of these distinctive lipids encompass sphingomyelin, ganglioside GM3, cholesterol, phosphatidylserine, disaturated lipids, and ceramide.^52^ Conversely, phosphatidylcholine and diacylglycerol are found in reduced quantities compared to the lipid composition of the originating cell membrane.^53^ Significantly, vesicles originating from MVBs display a higher concentration of phosphatidylserine facing the extracellular environment compared to the cellular plasma membrane. This characteristic may facilitate their uptake by recipient cells. Microvesicles have a lipid composition similar to their donor cell but are rich in polyunsaturated glycerophosphoserine and phosphatidyl serine. Microvesicles and exosomes contain higher phosphatidylserine in their membrane composition compared to the cellular plasma membrane. Whether they develop from MVBs or plasma membrane, these variations in lipid content across multiple kinds of vesicles indicate where they were biogenically generated.^54^

### Nucleic Acid Content

EVs contain a diverse genetic makeup, mainly genomic and mitochondrial DNA. However, they primarily contain short RNAs originating from various sources, such as ribosomal 18S and 28S rRNAs and tRNAs. Recent advances in next-generation sequencing identified a wide range of short RNA species within EVs, mainly short and long non-coding RNAs, tRNA fragments, vault RNA, piwi-interacting RNA, Y RNA, miRNAs, mRNAs, and rRNAs.^55^ The RNA length in EVs could be variable, where the majority of RNA is approximately 200 nucleotides, although a smaller fraction extends up to 4kb.^55^ Circular RNAs are also abundant and persistent in EVs.^56^ Once released into the extracellular environment, these RNAs within EVs appear to be protected from RNase degradation due to their encapsulation within the lipid bilayer membrane.^57^

## EVs-Associated Molecules and Targeting of Recipient Cells

The mechanisms by which EVs interact with cell surfaces and deliver their cargo to recipient cells are not yet fully understood. The initial interaction typically involves membrane-bound proteins and lipids present on the surface. The EV first docks onto the recipient cell plasma membrane, where it can remain attached, be internalized or fuse with the cell membrane. When EVs interact with target cells, receptors located on the plasma membrane such as integrins, can identify the cargo. This recognition event can initiate signaling pathways within the target cell.^23^ Binding to receptors can also promote the internalization of EVs through endocytosis, leading the EVs to enter endosomes and, in some cases, lysosomes for degradation. Mammalian EVs can also be internalized through receptor-mediated internalization, clathrin-mediated endocytosis, non-specific macropinocytosis, and endocytosis promoted by lipid rafts or caveolae.^23^

In order to transport their cargo, which consists of soluble proteins, nucleic acids, and eicosanoids into the cytosol of target cells, either the endosome membrane or the plasma membrane must be merged with EVs. This fusion process allows the transfer of EVs cargo into specific cellular compartments of the target cells, called vesicular fusion.^23^ Other ways of delivering EVs to the target cells are simple fusion with plasma membrane, or Golgi-mediated targeted delivery. In the former EVs can directly fuse with the plasma membrane of the recipient cells and deliver its content into the recipient cell cytoplasm. While in the latter, EVs can be taken up by the recipient cells and directed to the Golgi apparatus where they go through processing and sorting before their cargo is exported to specific cellular locations.^58^

**Figure 3:**
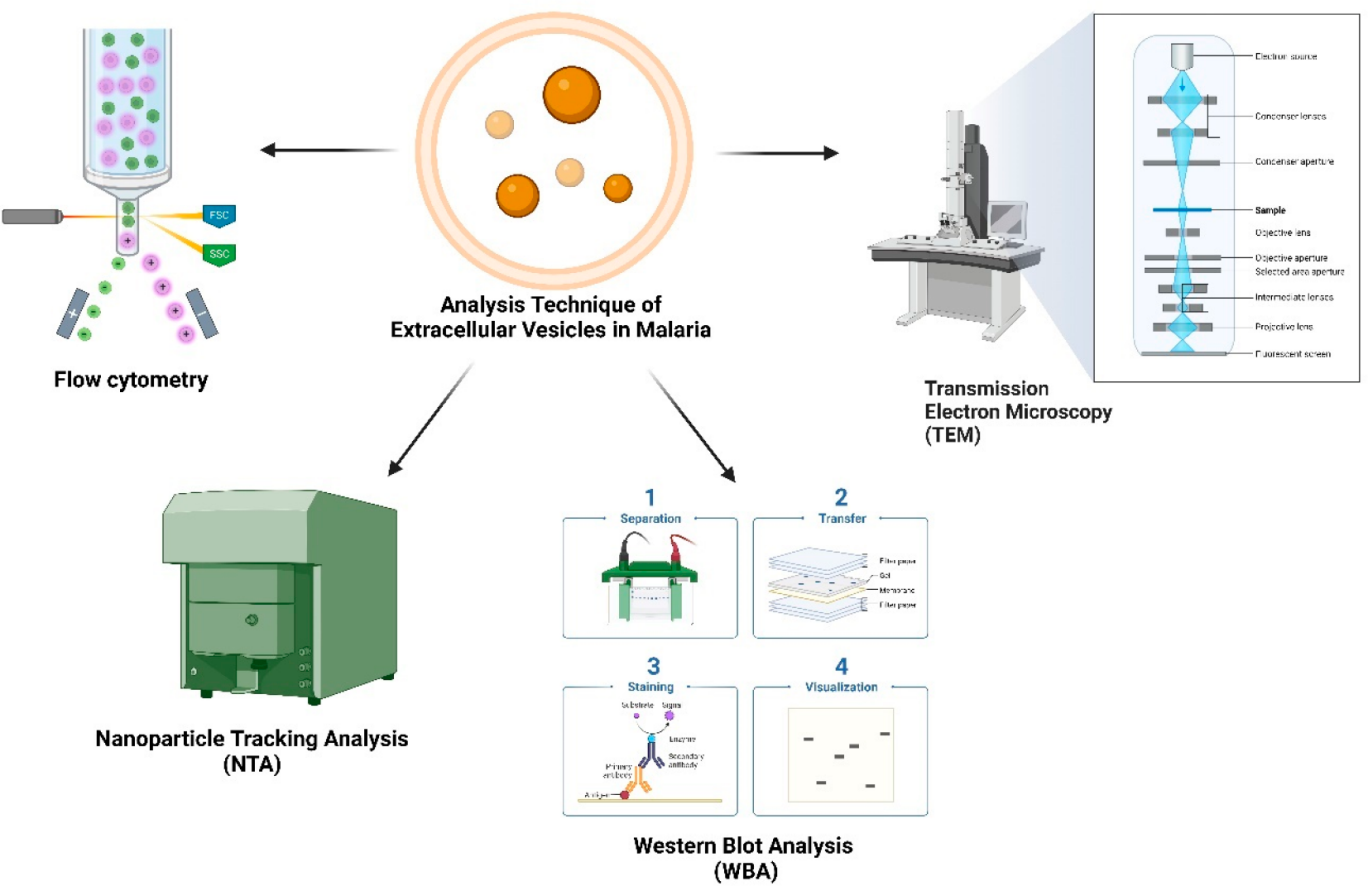
Employing advanced techniques—Flow Cytometry, Transmission Electron Microscopy, Nano Tracking Analysis, and Western Blot Analysis—reveals intricate details of extracellular vesicles during *Plasmodium*-infected malaria. These methods enable precise characterization, quantification, and profiling, providing essential insights into the dynamic interplay between extracellular vesicles and malaria pathogenesis.

Pathogen-derived EVs can be distinguished from mammalian-derived EVs by specific enzymes, toxins, or compounds containing pathogen-associated molecular patterns (PAMPs).^59^ These factors may account for differences in uptake and activity between EVs derived from pathogens and those from mammalian cells. Pathogen-derived molecules present in or on EVs could be utilized in biomarker studies and potentially have therapeutic applications. Hence, the precise mechanisms of EV–cell interaction and cargo delivery are still being investigated. The recognition of receptors, endocytosis processes, and fusion events play crucial roles in EV uptake and intracellular cargo transfer. Differentiating pathogen-derived EVs from mammalian-derived EVs based on their contents may have implications for understanding their uptake and potential applications in various fields.^60^ Utilizing sophisticated methodologies such as flow cytometry, transmission electron microscopy, nano tracking analysis, and Western blot analysis unveils intricate information about EVs in *Plasmodium*-infected malaria. These techniques facilitate accurate characterization, quantification, and profiling, offering crucial insights into the dynamic interplay between EVs and the pathogenesis of malaria (Figure 3).

## Role of EVs in Malaria Pathogenesis

EVs play a crucial role in facilitating host–pathogen interactions during malaria pathogenesis; however, *Plasmodium* species do not produce EVs.^61^ Advanced methods were used by Abou Karam and colleagues in isolating EVs to investigate and describe two separate subsets of EVs originating from RBCs that are infected with the protozoan parasite *P. falciparum*.^62^ These EVs, referred to as “Pf-iRBC-EVs”, have previously been implicated in the development of malaria-related clinical symptoms, including cerebral malaria.^63^ However, these EVs also play a significant role in facilitating cell-to-cell communication within the parasite, promoting the differentiation of *Plasmodium* into sexual forms. This process has important implications for the transmission of *Plasmodium* from human hosts to mosquito vectors.^61,64^

Diverse functional outcomes linked to malaria-derived EVs have not been fully understood, implying distinct roles for various EVs subpopulations. Recent studies have revealed that EVs produced by RBCs infected with *P. falciparum* at different stages of the parasite’s life cycle contain a variety of protein cargos, further supporting this notion.^61,65^ Recently, Abou Karam et al. (2022) have identified 132 proteins of which there were 23 *P. falciparum*-derived proteins along with 109 human proteins, including proteins that may promote the fusion of vesicles.^62^ There were 66 proteins that exhibited varying levels of abundance between the two EVs fractions: 6 *P. falciparum* proteins as well as 60 human proteins, suggesting that F3-EVs (30–70 nm) and F4-EVs (70–300 nm) contain distinct protein cargos that is inclusive of parasite as well as human-derived components. In the F3-EVs, there was a high abundance of complement-associated proteins, whereas F4-EVs were enriched with proteolysis-related proteins and proteasome subunits. This discovery provides insights into how different subsets of EVs can induce various phenotypic changes in the cells they interact with. This implies that each EV subgroup may follow various recipient cells or subcellular sites, where they may mediate distinct activities in the host–pathogen relationship and illness.^61,62^ When immune cells and vascular endothelial cells in the brain recognize parasite proteins on the outer layers of iRBCs or are released into the circulation as a result of burst iRBCs, it results in systemic inflammation in cerebral malaria.^66^

## Interaction of *Plasmodium*-Derived EVs and Host Red Blood Cells

EVs that are produced by parasites possess the ability to transfer virulence factors and drug resistance markers, modify the expression of host cell genes, and promote both parasite adherence and host cell proliferation.^67^ Throughout most of the parasite’s existence inside the vertebrate host, *Plasmodium* spp. inhabit RBCs, causing substantial changes in the structure and role of these cells. Additionally, *Plasmodium* spp. generate and release abundant proteins that can be found on the outer membrane of iRBCs.^68^ Research has shown that immune cells can be activated by EVs derived from *Plasmodium*-infected RBCs in cases of malaria. This facilitates communication between parasites and modifies various types of host cells, promoting infection.^11^ While some studies have shown that EVs promote inflammation, others have found that they suppress the immune system. Additionally, the content and characteristics of EVs may change depending on when they are released throughout the parasite’s development. For instance, only 12 h after red blood cell invasion do EVs include the parasite virulence component PfEMP1.^69^

## Interaction of *Plasmodium*-Derived EVs with Host Immune Cells

Natural killer (NK) cells play a crucial role in generating immune responses against malaria parasite infection and exhibit significant variations in their responses among individuals. In a recent study, it was discovered that NK cells with higher levels of the pathogen recognition receptor MDA5 (melanoma differentiation-associated gene 5) were more effective in initiating an immune response against iRBCs.^70^ These NK cell receptors are activated by EVs released by iRBCs. This implies that in the context of malaria infection, it may be possible to boost non-responsive NK cells by activating MDA5 receptors using EVs, offering a potential NK cell-centered intervention strategy for combating malaria infection in humans. However, the effects of EVs on the immune system are complex and can vary.^64,71^

Exposing human primary monocytes to EVs derived from parasites lacking PfEMP1 (a parasite virulence factor) results in the increased expression of genes associated with defense mechanisms. This suggests that EV-associated PfEMP1 plays a role in suppressing the immune response.^64,69^ The complexity of the immunomodulatory properties of malaria EVs, as well as the quantity and composition of host factors such as cytokines and chemokines generated during infection, may affect the overall influence of EVs on immune cells.^64^ As demonstrated in the instance of monocytes, iREVs have been hypothesized as key participants in a unique method of immune cell activation brought on by plasmodial molecules. Notably, monocytes actively internalize pf-iREVs containing nucleic acids.^11,72,73^ The EVs derived from *P. falciparum* contain native DNA cargo that is released into the cytosol of monocytes. Subsequently, the DNA cargo within these vesicles prompts the activation of STING (Stimulator of Interferon Genes), resulting in the release of type 1 interferons (IFNs) and various other cytokines.^11,73^

A cytosolic adaptor protein called STING is activated when DNA-binding proteins attach to it, playing a pivotal role in enabling innate immune cells to generate IFNs.^11,74^ Through imaging studies, it has been observed that upon transfection of *P. falciparum* DNA, monocytes exhibit the nuclear translocation of interferon regulatory factor 3 (IRF3), an activated transcription factor, confirming the method outlined by Sisquella and colleagues.^72^ Translocation of IRF3 is the last step in the STING-dependent signaling cascade before transcription of IFN genes takes place.^75^ The mechanism by which plasmodial DNA enters the cytoplasm of immune cells and triggers the STING-dependent innate immune response remains unclear.^11,76,77^ Although there is some debate over hemozoin–DNA binding,^78^ it has been proposed that the DNA can be delivered via hemozoin.^11,77^ Considering that disrupting the STING cascade has been demonstrated to enhance the survival of *P. berghei*-infected mice. These are prone to cerebral malaria, and a comprehensive exploration of the immune cell stimulation mediated by EV-DNA would offer valuable insights into the cerebral malaria pathogenesis and the involvement of IFNs.^11,77^

## Interaction of *Plasmodium*-Derived EVs with Host Endothelial Cells

The activation of endothelial cells is of paramount importance in the pathophysiology of *P. falciparum*-mediated malaria. This phenomenon arises from the selective attachment of mature-stage iRBCs to the microvascular endothelium in diverse tissues and organs, with a notable focus on the brain in the context of cerebral malaria.^79^ Endothelial cells have been shown to respond to a variety of stimuli.^80^ The release of EVs from TNF-activated endothelial cells in thrombotic diseases is one such *in vitro* response.^81^ This finding, combined with the awareness of elevated TNF levels in malaria, motivated the initial investigation into EVs originating from endothelial cells in the context of malaria.^82^*Ex vivo*, patient-derived endothelial cells from cerebral malaria, as well as non-cerebral malaria, have been demonstrated to react in different ways to TNF stimulation, with the former shedding considerably more EVs.^83^ Given the pivotal role of TNF in the progression of cerebral malaria, the distinct release pattern of EEVs in response to this pro-inflammatory cytokine is of significance. It calls for further research to explore the potential functions of EEVs in this disease. To gain a comprehensive understanding of the direct involvement of malaria-induced EEVs in the pathogenesis of cerebral malaria, it may be necessary to employ a comprehensive experimental model that combines *ex vivo*, *in vitro*, and *in vivo* components. This multifaceted approach can provide valuable insights into the specific role of EEVs in the development of this neurological condition.

## Host Immune Modulation by *Plasmodium*-Derived EVs: Pro-inflammatory and Immunomodulatory Effects

The process of membrane vesiculation, which involves the formation of membrane-bound vesicles, relies heavily on the ATP-binding cassette transporter A1 (ABCA1).^84,85^ Research has indicated that individuals suffering from uncomplicated or non-cerebral severe malaria, who possess genetic variations in the ABCA1 gene, exhibit notably reduced levels of EV release.^86^ On the other hand, patients with cerebral malaria or multiorgan dysfunction as well as the wildtype ABCA1 gene exhibit significantly higher EV release. The ABCA1 gene displays extensive genetic variation, and it may play a prominent role in the underlying molecular mechanisms responsible for the worsening of cerebral malaria through EVs.^87^ Recent studies using a rodent malaria model indicate that EVs could serve as a source of circulating parasite components. Animals with genetic or pharmacological impairments in EV synthesis do not develop cerebral malaria, further emphasizing the importance of EVs in the disease process.^13,15,64^

Second, during infections, EVs trigger a robust inflammatory response, leading to inflammation.^16,64^ Third, when EVs derived from the plasma of *P. berghei* ANKA-infected mice are transferred to another host, they disrupt the integrity of the blood–brain barrier.^14^ This disruption is associated with an elevated presence of tissue factor and phosphatidylserine in the circulating system, both of which are procoagulant molecules found abundantly on the membrane of EVs.^88^ High levels of circulating EVs contribute to coagulation and promote clot formation. Inhibiting the production of EVs through genetic means significantly reduces inflammation and mitigates the severity of the condition. *Plasmodium*-derived EVs might carry toxic molecules, contributing to cellular toxicity and affecting the function and integrity of host cells leading to tissue damage and organ dysfunction.^11,63,64^

The ABCA1 plays a key role in transferring phosphatidylserine across the cellular membrane to the outer leaflet,^64^ facilitating the external exposure of phosphatidylserine, which is a prerequisite for EV release.^64^ Experimental cerebral malaria studies have demonstrated a significant decrease in EVs in ABCA1 wild-type mice. *In vitro* experiments have shown that these EVs possess considerable procoagulant activity where potency increases as the concentration of EVs rises.^13^ ABCA1-deficient mice exhibit diminished phosphatidylserine exposure upon stimulation with Ca2+, as compared to wild-type mice. Prothrombinase activity and EV production assays have confirmed reduced phosphatidylserine levels after A23187 incubation in ABCA1-deleted mice compared to their wild-type counterparts.^85^ Moreover, when ABCA1 is overexpressed in cell lines, there is an increase in the release of cholesterol-rich EVs. In mice infected with *P. berghei* ANKA, ABCA1-deficient animals did not exhibit neurological symptoms and were completely protected against cerebral malaria.^89^ These ABCA1-deficient mice also showed reduced inflammation compared to wild-type mice, accompanied by significantly lower levels of TNF-α in the plasma.

Histological examination indicated a reduced occurrence of perivascular hemorrhages, and there were no indications of immune cell vascular sequestration in the ABCA1-deficient mice.^13^ Remarkably, a study discovered that variations in the human ABCA1 promoter were linked to the severity of malaria,^86^ suggesting its key role in modulating the levels of pro-inflammatory EVs during the disease. Furthermore, the pharmacological inhibition of EV generation using dietary provitamin pantethine, a critical regulator of lipid metabolism, prevents the development of cerebral illness in *P. berghei* ANKA-infected mice.^90^ An unadapted host response to parasite infection, characterized by a drop in circulating EVs and the maintenance of blood-brain barrier integrity, is thought to be the cause of the protective effect brought on by pantethine. Importantly, pantethine did not affect parasite growth.^15^ Pantethine and ABCA1 act via distinct routes, as pantethine therapy did not affect ABCA1 activity.^15^ Overall, these findings point to EVs having a pro-inflammatory and harmful function in the onset of neurological disorders.

The initiation of a robust pro-inflammatory type-1 immune response is associated with the manifestation of severe clinical symptoms in malaria. Notably, when macrophages were cultured *in vitro* with EVs derived from the plasma of *P. berghei*-infected mice, it led to the production of TNF-α and the expression of cell surface CD40. Surprisingly, these EVs proved to be a more potent activator of macrophage response *in vitro* compared to intact iRBCs.^16,64^ Similarly, EVs generated from *P. falciparum* iRBC *in vitro* triggered the release of IL-6 and TNF-α by human macrophages.^17^ Genetic or pharmacological inhibition of EV synthesis has been shown to protect against the development of cerebral malaria in live organisms. Conversely, the introduction of EVs through adoptive transfer into mice resulted in an exacerbation of neurological symptoms.^14,64^ Fluorescently labeled EVs derived from mice infected with *P. berghei* ANKA were administered to animals to monitor their fate *in vivo*. Remarkably, the EVs exhibited a swift disappearance from the bloodstream and were eliminated within minutes of administration.

Nonetheless, microscopic analysis of the tissues unveiled a peculiar observation. The study observed that EVs were trapped along the endothelium within the brain arteries’ lumen in infected animals, whereas they were absent in healthy recipient mice. This implies that EVs have a role in priming the immune system for a pro-inflammatory response. These discoveries strongly indicate that EVs are implicated in the initiation and exacerbation of severe malaria by provoking a vigorous pro-inflammatory reaction.^64^ EVs produced from parasites are thought to play a more direct role in the immune-related processes connected to cerebral malaria, according to new laboratory research. Specifically, these parasite-derived EVs, known as pf-iREVs, prompted human monocyte-derived microglia to release higher levels of TNF-α while simultaneously reducing the production of IL-10.^11,91^ Therefore, the finding that pf-iREVs elicit an immune-modulating reaction in microglia aligns with the observations made by Mantel and their research team.^11,17^*In vitro*, laboratory experiments revealed that *P. falciparum*-infected pf-iREVs prompted the secretion of pro-inflammatory cytokines and increased the expression of vascular cell adhesion molecule 1(VCAM1) in human endothelial cells. However, the most notable finding was the presence of host-derived RNA-induced silencing complexes (Ago2-miRNA) within pf-iREVs, which directly influenced the functions of the endothelial barrier.^11,92^ Due to the biomolecular cargo they transport, these findings strongly indicate that iREVs have a role in promoting immunopathology and vascular dysfunction in the context of malaria.^11^

## EVs-Mediated Effects in Malaria Pathogenesis

Malaria parasites utilize EVs to manipulate the host immune system for their survival. The proteins, lipids, nucleic acids, and glycans present in pathogen-derived EVs have been associated with pathogenic effects on the host immune system.^59^ Research suggests that the parasite exploits EV secretion in iRBCs, altering the composition of released cargo. These EVs are enriched with parasite surface antigens and proteins associated with immunosuppression.^17^ Recent findings indicate that EVs from *P. falciparum* contain the parasite’s genomic DNA, which can activate cytosolic innate immune cell receptors in the host upon uptake of these EVs.^73^ EVs, including exosomes, microparticles, and microvesicles, are spontaneously released and their release is enhanced under cellular stress.^93^ These small vesicles, not only released by iRBCs but also by other cell types, are believed to play a role in malaria pathogenesis, along with other factors such as GPI anchors and hemozoin.^94^

## EVs-Mediated Cellular Communication and Immunomodulation

Living organisms have evolved various mechanisms of cell-to-cell communication to increase their chances of survival and development. These communication methods include the release of soluble signaling molecules, direct interaction between cells, and the transmission of signaling cargo through EVs. Several studies involving humans have demonstrated that the levels of circulating EVs derived from various cell types increase during *Plasmodium* species infection, and the severity of the disease is correlated with the plasma levels of these EVs.^95^ iRBCs of *P. falciparum* transfer DNA within the parasite population through exosome-like EVs. These EVs, released by *P. falciparum*-infected RBCs, facilitate the transfer, reception, and dissemination of information that is advantageous for the expansion of the parasite, regardless of whether the environment is stressful or not. The presence of functional payloads within EVs, which can be transferred from donor to recipient cells, has sparked interest in studying cellular communication.

Studies have revealed that EVs carry functional miRNAs (Table 1) and mRNAs. Moreover, EVs house a diverse array of other small non-coding regulatory RNA molecules, encompassing vault RNA, structural RNAs, Y RNA, tRNA fragments, and small interfering RNA.^96^ Malaria EVs include functional human miRNAs,^92,97^ the most abundant of which is miR451a. This miRNA is necessary for the concluding phases of RBC maturation.^98^ The mature form of miR-451a is located within EVs, where it combines with Argonaute-2 to create an RNA-induced silencing complex (RISC).^64,99^ The EV-transferred miR451a was directed at genes implicated in vascular permeability. Consequently, the increased levels of EVs observed in the plasma of severe malaria patients could potentially influence the integrity of the blood-brain barrier by transporting miR-451a to the endothelium.^100^ Malaria EVs also include plasmodial genomic DNA (gDNA).^64,73^ EVs facilitate the transfer of plasmodial gDNA from parasites to human monocytes. Once inside the monocytes, this gDNA activates STING, an immune-related adapter present in the cytoplasm of innate immune cells, responsible for detecting microbial DNA. Upon internalization of EVs, the gDNA of *P. falciparum* is released into the cytoplasm of the host cell. This leads to the recognition of DNA by STING, triggering a pro-inflammatory response through the secretion of cytokines.^64,73^

**Table 1:** EVs-Associated Non-coding RNAs in *Plasmodium* Infection

	**ncRNA name**	**Regulation**	**Comments**	**Ref**
1	miR-150-5p	Upregulated	Plasma sample from *P. vivax*-infected patients	(101)
2	miR-15b-5p	Upregulated	Plasma sample from *P. vivax*-infected patients	(101)
3	miR-451a	Upregulated	Individuals infected with *P. vivax* had lower levels of miR-451a expression than *P. falciparum*-infected patients	(102)
4	miR-16	Upregulated	Individuals infected with *P. vivax* had lower levels of miR-16 expression than *P. falciparum*-infected patients	(102)
5	miR-146a	–	Contribute to innate immunity in malaria during pregnancy	(103)
6	Let-7a-5p	Upregulated	Plasma sample from patients infected with *P. vivax* as well as *P. falciparum*	(101)
7	miR-486	–	Identified in iREVs and uREVs (uninfected red blood cell-derived EVs)	(104)
8	miR-181a	–	Identified in iREVs and uREVs	(104)

**Figure 4:**
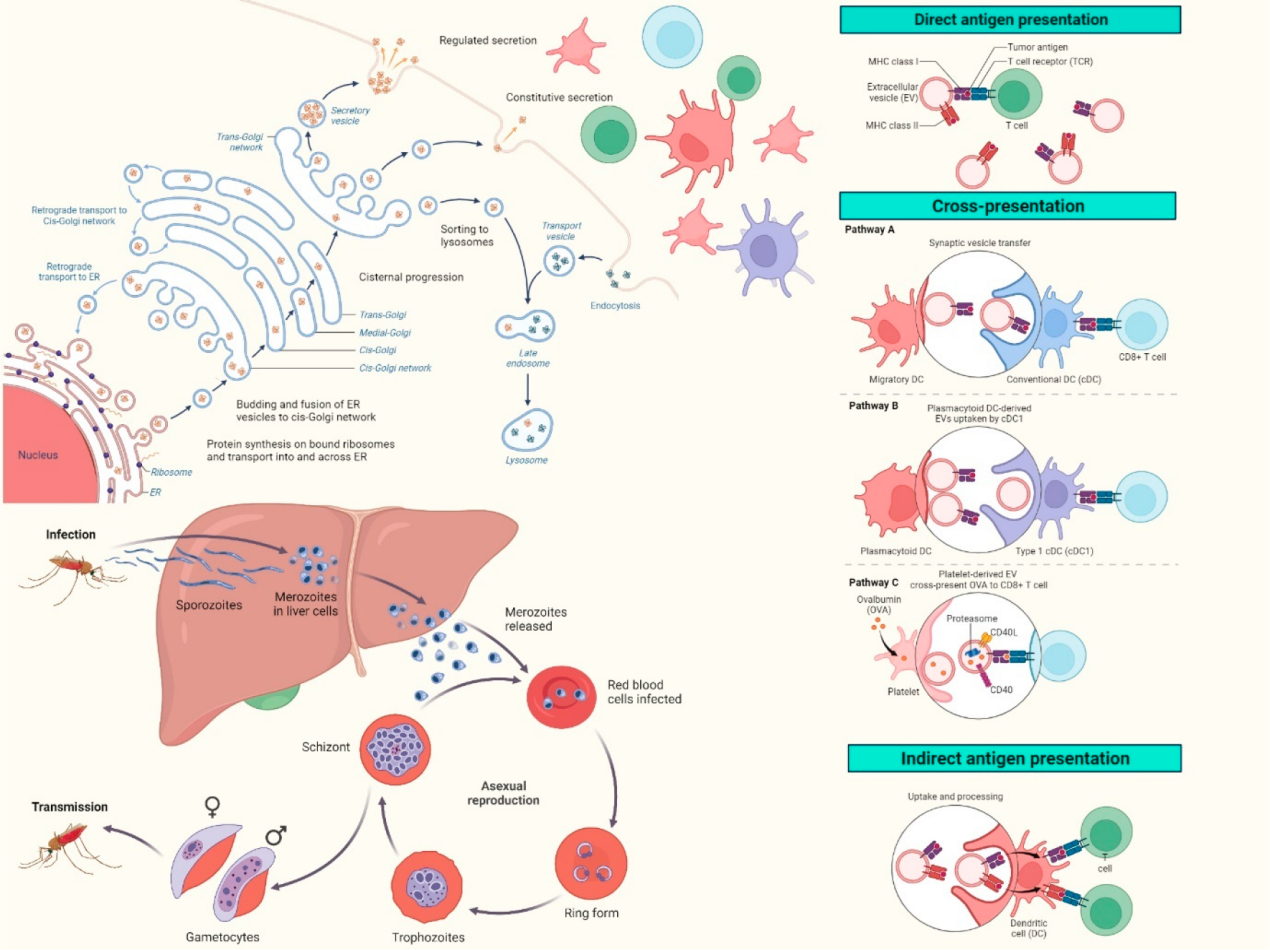
In malaria, diverse extracellular vesicles (EVs) play pivotal roles through distinct antigen presentation pathways. Direct antigen presentation involves EVs directly displaying parasite antigens, priming immune responses. Cross-presentation extends this impact, as EVs transfer antigens to different antigen-presenting cells, amplifying immune recognition. Indirect antigen presentation involves EVs modulating host cells to present antigens, influencing immune responses. This intricate interplay shapes the host–pathogen dynamics during *Plasmodium* infection. Understanding these EV-mediated antigen presentation mechanisms is crucial for unraveling the complexities of malaria pathogenesis and devising targeted therapeutic strategies. The triad of direct, cross-, and indirect antigen presentation by EVs reveals the multifaceted nature of host–immune interactions in response to *Plasmodium*, offering insights into potential intervention strategies for this globally significant infectious disease.

The activation of endothelial cells and the subsequent adherence of iRBCs to the endothelium in the brain’s microvasculature can lead to vascular dysfunction. This can be accompanied by the accumulation of cellular clusters consisting of both uninfected RBCs (uRBCs) and infected RBCs, causing blockages in small blood vessels, and damage to the walls of the blood vessels. As a result, microhemorrhages can occur, and the levels of pro-inflammatory chemokines and cytokines in the bloodstream can rise.^66^ The micro bleedings allow parasite products to come into contact with brain resident cells. The rodent parasites efficiently transmit EVs to astrocytes and, to a lesser extent, microglia. Activation of EVs leads to an increase in interferon-gamma inducible protein 10 (IP10), a pro-inflammatory cytokine known to be associated with the recruitment of pathogenic CD4 and CD8 T cells into the brain, as well as contributing to the pathophysiology of rodent malaria^64,105^ (Figure 4).

Under stressful conditions, such as exposure to antimalarial drugs, the synthesis of exosome-like vesicles is significantly increased, providing a means for the parasite to adapt to its environment. In the case of *P. falciparum*, this increased production of vesicles is linked to the differentiation of sexual forms and the parasite’s ability to evade the mosquito vector under unfavorable conditions for the host’s survival. The discovery of the *P. falciparum* protein PfPTP2, involved in intercellular communication, suggests that these exosome-like vesicles originate from Maurer’s clefts rather than from the membranes of RBCs. The particles coated with PfPTP2, found within *P. falciparum*-infected RBCs, bear a resemblance to vesicular structures known as electron-dense vesicles (EDVs) or may be connected to other particles referred to as J-dots.^106,107^ The presence of PfPTP2-coated vesicles during their formation from Maurer’s clefts suggests that these vesicles originate from these large vesicular structures, which have essential roles in sorting, targeting, and packaging proteins. These structures share similarities with late endosomes, known for their involvement in intracellular transport. Additional research is necessary to ascertain whether the PfPTP2-coated vesicles are indeed identical to MVBs and if exosome-like vesicles are directly derived from MVBs through secretion across the plasma membrane of RBCs. The disruption of PfPTP2 function results in a decrease in the quantity of extracellular exosome-like vesicles, reinforcing the notion that PfPTP2-labeled vesicles play a vital role in the formation and release of these exosome-like vesicles.^106^ It is also clear that PfPTP2 is necessary for the target cell to receive signals.

The literature demonstrates that *P. falciparum*-infected RBCs produce exosome-like vesicles that enable drug-treated parasites to survive and result in a much higher number of gametocytes that can transfer to the next host.^106^ In Malawi, it was observed that *P. falciparum*-infected children had elevated levels of endothelial cell-derived EVs in their plasma compared to healthy control subjects.^82^ In a different research study, the levels of RBC-derived EVs were compared among individuals infected with *P. vivax*, *P. malariae*, or *P. falciparum*. It was observed that individuals with severe malaria caused by *P. falciparum* had the highest levels of EVs.^108^ Although these studies do not establish a direct causal relationship between elevated EV and severity of disease, *in vitro* experiments and research conducted on mice indicate that EVs originating from platelets as well as endothelial cells play a role in the overall inflammatory response associated with malaria.^16^ Intriguingly, inflammatory reactions boost parasite sequestration by activating receptor expression in endothelial cells.

The mouse malaria model also verifies RBC-derived EVs’ pro-inflammatory features, since those purified from infected mice significantly activate macrophages in a TLR-dependent manner *in vitro*.^16,109^ According to a recent study, macrophages quickly absorb EVs produced from the iRBC supernatant. This absorption triggers a potent immune response that is characterized by the release of cytokines that are both pro- and anti-inflammatory.^17^ This discovery raises questions about the mechanism by which iRBCs, which do not possess internal membranes or the necessary machinery for exocytosis and endocytosis, can release EVs. Through detailed ultrastructural investigations, it has been observed that malaria parasites profoundly alter the environment of the host RBC.^110^ This alteration includes the establishment of a protein network that facilitates the nutrient intake and export of virulence factors.^111^

Recent research has revealed the presence of numerous proteins that the parasite exports, traversing its membrane and extending beyond the parasitophorous vacuole membrane into the host cell. These findings shed light on the complex mechanisms employed by malaria parasites to interact with the host and manipulate immune responses. A specific subgroup of these exported proteins, which includes the parasite solute transporter Clag3 proteins along with the primary virulence factor PfEMP1, is transferred and deposited onto the surface of RBCs.^112^ The transportation of proteins to the surface of iRBCs is facilitated through parasite-induced membrane structures called Maurer’s clefts, which are firmly attached to the host cytoskeleton.^113^ Tiny vesicles linked to actin filaments are transported back and forth between Maurer’s clefts and the membranes of RBCs. Remarkably, these structures are absent in individuals with sickle cell hemoglobin mutations, suggesting a possible mechanism for protection against malaria.^109^

Proteomics analysis revealed that in EVs derived from iRBCs, there is an increased presence of host lipid raft proteins, including stomatin, as well as the markers of microvesicles like ARF-6 and VPS4. This suggests that only a small fraction of host machinery is involved in EV synthesis. Additionally, several parasite proteins, particularly those originating from Maurer’s Clefts structures, were identified in the EVs. Notably, when a specific component of Maurer’s Clefts was deleted, EV synthesis and uptake were suppressed.^106^ Quantitative and time-based evaluations of EV releases demonstrated that iRBCs produce approximately ten times as many EVs as uRBCs. The majority of these EVs are released just before the parasite egresses from the RBCs.^17^ The observed absence of Maurer’s Clefts and the time of its release are related, as observed in recent studies that analyzed the development of parasites over time.

It is interesting to note that iRBCs may internalize EVs and transmit them into the cytoplasm of the parasite, indicating an intriguing function for them in intercellular communication between parasites. The presence of additional membranes enclosing internalized EVs indicates the involvement of phagocytic or endocytic processes within iRBCs, which are likely facilitated by the parasite itself.^17^ The binding and internalization of EVs by iRBCs can be adjusted according to the concentration of EVs, and this process correlates with the enhanced development of malaria transmission stages, particularly gametocytes. These results suggest that the parasite population uses EVs as a cellular communication channel with density-sensing capabilities.^17^

Moreover, it has been noted that EVs originating from transgenic parasites can transfer DNA containing a marker for drug resistance among individual parasites. This raises the possibility of propagating drug resistance throughout the parasite population.^106^ These resistant parasites are involved in the transmission stage of the malaria parasite’s life cycle, suggesting the existence of a cellular communication mechanism. These discoveries support earlier observations that parasite-conditioned media, the source of the EVs used in the experiments mentioned, can stimulate the production of gametocytes. Therefore, further investigation into this newly discovered cellular communication pathway is warranted.^114^

Cumulatively, the existing body of research provides evidence that *P. falciparum* parasites have developed a distinctive method of intercellular communication using EVs to sense their population density during an infection. This adaptive process enables them to manage the delicate equilibrium between virulence, represented by asexual replication, and transmission, which entails the production of gametocytes. These findings highlight the communication abilities of *P. falciparum* parasites, enabling their survival and promoting the enhanced differentiation of gametocytes for disease transmission.^106^

## EVs in Developing Novel Therapeutics against Malaria

One of the key challenges in managing malaria is the development of drug resistance, which arises from the parasite’s exposure to suboptimal drug levels. This challenge is compounded by various factors: (i) the complex life cycle of the *Plasmodium* parasite, making it difficult to effectively target and eliminate^115,116^ (ii) the physical conditions within the circulatory system, marked by strong fluid flow, resistance, and shear forces, impact the way drugs interact with target red blood cells, and (iii) the challenging aspects of antimalarial drugs, which often possess amphiphilic properties, contribute to their challenges. These drugs have a wide distribution throughout the body, which leads to rapid metabolism in the liver and relatively short half-lives, often ranging from a few hours to less than 1 h.^117^ In this circumstance, the development of more effective malaria therapies is critical. Antimalarial drug administration typically takes place within a narrow therapeutic window whereby excessive dosages result in negative side effects and inadequate local concentrations result in resistance.^116,117^ To mitigate drug resistance, the current recommendation is to administer combinations of two drugs or more, that operate through different mechanisms of action and target separate biochemical pathways within the parasite.

EVs have developed as potentially effective drug delivery strategies for specific organs or cells.^118−120^ EVs have several benefits over synthetic delivery systems, which include (i) greater blood stability due to their natural surface composition^118,120^ (ii) a proteo-lipid architecture may provide superior cargo protection,^118^ (iii) they have surface ligands and adhesion molecules that give endogenous cell and tissue targeting properties,^118^ (iv) an improved biocompatibility better permeability through biological barriers, such as the blood–brain barrier,^121^ and (v) nearly non-immunogenic properties when derived from autologous sources.^118^ EVs derived from several cell types have been employed to transport a variety of medicinal substances,^122^ including macromolecules (DNA, RNA, and proteins).^123^ The researchers employed various techniques to assess the size and composition of EVs derived from iRBCs and uRBCs. Furthermore, they loaded antimalarial drugs into EVs and evaluated their effectiveness in inhibiting parasite growth.^118^

EVs offer numerous potential benefits. They are relatively easy to utilize and offer a wide range of options for surface engineering and cargo encapsulation.^124^ They have been implicated in providing therapeutic effects against various disorders related to membrane defects, and compounds attached to the EV surface have demonstrated targeting capabilities, increased expression levels, enhanced solubility, and improved antigen immunogenicity.^125^ Compared to relevant viruses, EVs are more biocompatible as they have evolved mechanisms to evade or resist infection by the human immune system. The primary benefit of EVs is their capacity to offer an optimal membrane environment for membrane proteins, enhancing both their dynamic behavior and stability.^126^

Researchers used a unique method to assess the potential of EVs made from *P. falciparumin vitro* cultures, also known as iREVs, and compared them to EVs made from uRBCs, or uREVs (uninfected red blood cell-derived EVs), as possible natural carriers for delivering antimalarial medicines.^118^ iREVs exhibited significantly higher binding affinity to both iRBCs and uRBCs compared to uREVs. This innovative targeted drug delivery system shows great promise for enhancing antimalarial therapy and addressing drug resistance.^118^ iREVs demonstrated strong uptake by numerous cell types, consisting of endothelial cells, T cells, monocytes, glial cells, and NK cells, suggesting their potential in the development of adjuvant immunotherapies for cerebral malaria.^118^ Moreover, iREVs involve exploiting their inherent mechanism to specifically target host cells. iREVs carry surface molecules that can specifically recognized by receptors on the surface of host cells, facilitating their uptake. The use of parasitic EVs may also lead to cellular tropism, which means preferential targeting of host cells.

NK cells serve a crucial role in the initial response to malaria parasite infection by rapidly activating cytotoxic activity and cytokine production. However, during acute malaria, NK cell cytotoxicity is often reduced. The uptake of iREVs by NK cells presents an intriguing prospect for anti-malarial therapy and warrants further investigation. Laboratory experiments conducted *in vitro* have demonstrated that NK cells from individuals with severe malaria exhibit reduced effectiveness in inhibiting the growth of *P. falciparum* parasites. This may be attributed to immune suppression during the disease or a state of “hyper-immune” response that eventually leads to functional exhaustion of NK cells.^70^ In co-culture experiments involving pf-iREVs, NK cells, and iRBCs, the presence of iREVs transformed a “non-responsive” population of NK cells into a “responsive” one, resulting in a reduction in parasitemia levels.^71^

Patients affected with severe malaria have been associated with an abundance of “non-responding” NK cells.^71^ The RNA content of iREVs has been linked to the increased activity of NK cells. MDA5 activation by RNA from iREVs was observed to activate NK cells, as indicated by a notable rise in the expression of the activation marker CD69. However, there was only modest stimulation of IFN production.^71^ MDA5 functions as an intracellular sensor for foreign RNA and its activation is crucial for initiating an innate immune response that can impact the outcome of malaria infection.^127^ The researchers proposed the hypothesis that the RNA from the malaria parasite, which is encapsulated within EVs, is released into the cytosol upon the fusion of EV and NK cell membranes.^71^ The study’s findings also indicated that iREVs could indirectly activate NK cells by facilitating direct physical contact between iRBCs and NK cells. This was accomplished by inducing a high-affinity interaction involving lymphocyte function-associated antigen 1 (LFA-1).^128^ Such direct interactions between iRBCs and NK cells are essential for the efficient production of interferon against the *Plasmodium* parasite. If it is possible to identify the particular RNA species in iREVs that activate and prime NK cells, it may offer the potential to develop natural EVs containing targeted functional RNA as a future NK cell-based therapy for malaria.^71^

Conversely, it has been demonstrated that the presence of functional human miR-451 along with miR-140 complexes in EVs originating from uRBCs exerts a harmful influence on the pathogenicity of *P. falciparum*.^104^ Infection with malaria leads to the release of EVs from uRBCs. These EVs transport hmiR-451 and hmiR-140, which, upon uptake by iRBCs. This reduces the expression of genes responsible for encoding PfEMP1, which is a virulence protein. Furthermore, both iREVs and uREVs impede the invasion of RBCs by merozoites.^104^ In contrast to the previously discussed pathophysiological impact of iREV and hmiRNA complexes on host endothelial cells, the hmiRNA complexes in uREVs have a negative effect on parasite survival.^92^ This highlights the complexity of malaria and underscores the importance of thorough research. Such investigations should encompass clinical isolates of *P. falciparum* as well as rodent malaria parasites to elucidate the intricate interactions between iREVs, uREVs, and the vascular endothelium. Moreover, these investigations should seek to elucidate the factors that dictate whether these vesicles have a harmful or advantageous impact *in vivo*.^129^

Meanwhile, host RBC-derived miRNAs offer potential as a novel malaria medication due to their capacity to inhibit parasite survivability and reduce parasitemia^104^ and that can also be given in EVs. In recent studies, it has been discovered that the bloodstreams of individuals with malaria contain a significant presence of microparticles. Through their findings, researchers have identified that miR-451/140 actively suppresses the expression of a crucial malaria antigen called PfEMP1. This inhibition is achieved by the binding of miR-451/140 to the A and B subgroups of var. genes, which are responsible for encoding PfEMP1. Furthermore, it has been observed that mature RBCs contribute to innate resistance against malaria infection by releasing MPs.^104^

## EVs in Developing Novel Vaccines against Malaria

The development of a successful vaccine has proven to be a formidable task, primarily due to the antigenic variability and intricate life cycle of *Plasmodium* species, the parasites responsible for causing malaria. Research on iREVs has shed light on new pathways in malaria and identified potential therapeutic targets, as well as cargo carried by host-derived EVs that have anti-malarial properties. Furthermore, parasitic molecules associated with EVs are being tested in vaccination trials. Indeed, a handful of studies have showcased the potential of employing complete EVs derived from malaria as candidates for vaccine development.^11^ Demarta-Gatsi and their team have recently shown that mice with no prior exposure to malaria when administered with iREVs obtained from mice infected with *P. berghei*, not only survived an initial severe infection but also established durable immunological memory that conferred immunity against a subsequent infection.^130^

The researchers speculated that genetic factors might have influenced the survival rates, as different mouse strains exhibited varying rates of survival. They also discovered that EF-1, an immunogenic protein associated with EVs, played a role in the immune response. Mice that received immunization with recombinant EF-1 also managed to survive the infection, but the clearance of parasites took longer compared to mice immunized with iREVs containing EF-1. In the experimental malaria model using *P. berghei*, histamine-releasing factor (HRF) was found to have a significant immunomodulatory effect, and its deletion resulted in a long-lasting protective immune response in mice.^130^ HRF interacts with EVs and, together with another vesicle component called elongation factor 1 (EF-1), suppresses antigen-specific T-cell responses by interfering with the critical phosphorylation pathway. These pathways are associated with TCR signaling.^130^

*Plasmodium* parasites use immunosuppressive EVs as part of their effort to evade the immune response triggered by the host. Moreover, this discovery will have an impact on malaria vaccine development aimed at long-term anti-parasite immune responses.^130^ While not explicitly mentioned in the article, this implies that immune response-eliciting EVs derived from infected reticulocytes are more immunogenic and better suited for targeted administration of immunogens. In a controlled malaria experiment, mice that were vaccinated with EVs derived from reticulocytes infected with non-lethal*P. yoelii*, along with the addition of CpG-ODN adjuvant, demonstrated a targeted humoral immune response against the parasite.^12^ Furthermore, in an experimental malaria model, this immune response led to the activation of effector memory T cells within the spleen.^131^ In the end, the mice were immunized, which resulted in their protection against both initial and subsequent lethal *P. yoelii* infections. Examination of EVs originating from *P. yoelii*-infected reticulocytes uncovered the existence of approximately 70 *Plasmodium* proteins. Among these proteins were notably immunogenic rhoptry proteins and merozoite surface protein 1 (MSP1).^132^ These discoveries indicate that malaria-derived EVs have the potential to act as antigen-presenting entities, presenting an exciting avenue for the malarial vaccine development. Previous experiments in malaria vaccine research have predominantly centered around the use of synthetic nanoparticles and microparticles for delivering parasite DNA or proteins. Among these approaches, the biocompatible polymer named poly(lactic-*co*-glycolic acid) (PLGA) has been prominently highlighted.^133^

Shan Liu and colleagues have focused their research on employing a distinctive ultrasonic atomization technique to produce PLGA microparticles loaded with concentrated malaria plasmid VR1020-MSP119.^132^ This technique, known for its ease of formulation and operation, as well as its consistent microparticle synthesis, proves to be commercially feasible for manufacturing biodegradable microparticles consisting of condensed malaria pDNA molecules. Their research marks an initial step toward the ultimate objective of producing microparticles on a large scale for clinically effective malaria pDNA vaccines.^132^ The significant involvement of EVs in malaria opens up possibilities for utilizing them as a delivery system for malaria vaccines. By encapsulating synthetic EVs within nanovesicles, it may be possible to develop a promising therapeutic approach for the creation of anti-malarial vaccines.

Furthermore, *in vivo*, experiments in mice have shown that PLGA-coated iRBC-derived EVs have immunization capability.^12^ In contrast to conventional vaccination methods, utilizing PLGA as a carrier for delivering *P. vivax* antigens demonstrated a favorable and improved immune response.^134^ Additionally, *Plasmodium* antigen-encoding plasmid DNA has been delivered to antigen-presenting cells using PLGA microparticles.^132^ Transmission-inhibiting vaccinations that target the parasite’s sexual stage have been demonstrated to be promising yet inefficient. Controlled gradual release of loaded antigens in biodegradable microparticles, on the other hand, has been shown to produce prolonged functional antibody responses, potentially making the anti-malaria vaccine more powerful.^133^

Recently, in a Phase III clinical trial, the RTS,S vaccine showed limited efficacy during a seven-year follow-up among young African children.^135^ As a result, there is considerable interest in leveraging the potential of EVs to improve vaccine delivery. Cutting-edge technologies for the large-scale production of EVs, such as exosome-mimetic nanovesicles, show potential as a viable therapeutic approach for the creation of anti-malarial vaccines. The sexual stage of mosquitoes and parasites can be targeted with the help of transmission-blocking vaccines. This could not be achieved due to a lack of natural antigen presentation in the human host.^133^ Another way that can make the vaccine more effective is by using biodegradable microparticle packaging of antigens. The advantage of this is that acts by slow release of antigens thus gaining long-lasting functional antibody responses.^133^ This vaccination strategy can be improved with additional lipid vesicle manipulation and better adjuvants to allow for increased humoral immunity and vaccine potency.^136^ Thus, the most effective method of malaria vaccination may be a combination vaccine that contains top-candidate antigens and is administered via microparticles/EVs or mimetic nanovesicles.

## Emerging Role of EVs in Malaria Treatment

The finding that exosomes produced by parasites can spread genes for drug resistance has revealed previously unanticipated possible methods for horizontal gene transfer in populations of wild parasites and may be especially pertinent given the current situation with developing drug resistance.^63,137^ Additionally, EV may work well as malaria vaccine delivery systems. Despite extensive research efforts spanning several decades, developing an effective vaccine against *Plasmodium* remains a significant challenge in the scientific community. However, the promising outcomes observed in the delivery of leading vaccine candidates using EVs and microparticles encourage further investigation in this area. Further research is required, but indications are suggesting that *Plasmodium* parasites might actively manipulate the host using vesicles. Human immune cells have been reported to recognize and react to *Plasmodium* microvesicles as immunostimulatory agents, and EVs from iRBCs may contain functional microRNA that could impact endothelial cells.^17,63^ More specifically, the possibility that *Plasmodium* could employ EVs to modulate the host immune system is of significant interest. Conversely, these vesicles could also interact with host RBCs, modifying or preparing them for parasite invasion, thus creating a more permissive environment for successful infection. This still needs not to be set in stone and is a region that requires further examinations.^63^

## Conclusion

The network of EVs in malaria involves intricate communication at both molecular and cellular levels, facilitating interactions between different cells of the host and various parasite populations, and interactions between host cells as well as parasites themselves. The relationship between EVs found in the bloodstream and the severity of the disease in natural infections, along with evidence from gene knockouts and drug interventions protecting against cerebral malaria, indicates that EVs hold the potential to function as biomarkers and imply their involvement in the development of the disease. EVs originate from different cell types, such as endothelial cells, erythrocytes, and platelets, and it has been observed that their levels increase significantly during severe infections. This implies that EVs could potentially play a role in promoting inflammation and influencing the overall progression of malaria pathogenesis. Throughout evolution, the malaria parasite has developed an optimized mechanism for infecting and surviving in hostile environments.

One crucial aspect of this mechanism is the production of small vesicles by iRBCs, which play a vital role in facilitating communication and coordination among the parasites, enabling them to differentiate into gametocytes that can be subsequently acquired by mosquitoes. EVs play a role in regulating the immune system by transporting various molecules, including RNAs, from iRBCs to immune cells. Depending on the specific cellular environment, this can result in either immune suppression or immune activation. The scientific community has extensively studied the involvement of EVs, whether derived from the host or the malaria parasite. In particular, EVs originating from the host are implicated in the progression of severe malaria. Many studies have focused on using artificial microparticles/microspheres like PLGA as carriers for malaria vaccines. Administering PLGA vesicles loaded with *P. vivax* antigens (such as merozoite surface protein-1, apical membrane antigen-1, or serum sporozoite protein) via the intranasal mucosa has shown to elicit more potent immune responses, both in terms of humoral and cell-mediated immunity, compared to conventional adjuvanted vaccines. This demonstrates the potential of utilizing PLGA vesicles as an improved immunization strategy.

## Limitation of EVs Study in Malaria

With substantial molecular and cellular interaction among several host cells, within parasite populations, and between host cells and parasites, the EV network in malaria is intricate. The possibility of EVs as biomarkers is supported by the relationship between circulating EVs and disease severity in natural infections as well as the protective effects of gene knockouts and pharmacological inhibitors against ECM. These findings also indicate a harmful function for EVs in disease. Certain EV populations have also been suggested to have a protective role. The biomolecular cargo of EVs confers upon them a multitude of characteristics. The biology of malaria EVs is increasingly being understood, but there are still many unanswered questions regarding the unique characteristics of the EVs that are released at different stages of the parasite’s life cycle and how these may differ between individuals. Furthermore, it is yet unclear how specialized cargo loading, EV release, and EV uptake function. A better understanding of the pathogenic and protective pathways of EVs is imperative for the effective utilization of EVs as vaccines and therapies that inhibit or stimulate these pathways.

## Future Prospects and Challenges

A lot of scientific research is needed to be done on the possibility that EVs could be utilized to develop malaria vaccinations. Also, it is necessary to make technological advancement to enable a large-scale separation of EVs from *Plasmodium* culture and the generation of homogeneous EV populations. However, it is yet unclear what functions these EVs might have during human active infection and what global modulatory impacts they have on the host. More studies should be conducted to characterize different malaria EV populations, as well as to examine their biogenesis, destiny, and potential roles in the disease. The unique characteristics of EVs released by the various stages of malaria parasite life, as well as how this may differ from person to person, are still largely unknown. Deeper comprehension of the pathogenic and protective pathways of EVs is imperative for the effective utilization of EVs as vaccines and therapies to impede or stimulate these pathways.

A multifunctional strategy is necessary to address the challenges of researching and fighting malaria in different geographic regions, especially in nations like Nigeria, which currently has the highest malaria rate. The emergence of drug-resistant parasites is a major worry that requires coordinated efforts to stop. There are several obstacles in other countries like Africa, South Sudan, and Nigeria, including unhygienic circumstances and the urgent need to raise public awareness of the disease. Making individuals more aware of preventive measures and guaranteeing their commitment to these methods are crucial stages in spreading awareness about malaria. Another significant obstacle is funding, since developing and producing antimalarial medications can be an expensive process. Resources are difficult to obtain and allocate for various uses, necessitating international cooperation and strategic planning. All things considered, resolving the issues caused by malaria in various geographic settings necessitates an integrated and well-coordinated approach that includes public awareness campaigns, innovation and research, legislative actions, and significant financial investments.
